# Genes essential for the morphogenesis of the Shiga toxin 2-transducing phage from *Escherichia coli* O157:H7

**DOI:** 10.1038/srep39036

**Published:** 2016-12-14

**Authors:** Shakhinur Islam Mondal, Md Rakibul Islam, Akira Sawaguchi, Md Asadulghani, Tadasuke Ooka, Yasuhiro Gotoh, Yasuhiro Kasahara, Yoshitoshi Ogura, Tetsuya Hayashi

**Affiliations:** 1 Division of Microbiology, Department of Infectious Diseases, Faculty of Medicine, University of Miyazaki, 5200 Kihara, Kiyotake, Miyazaki 889-1692, Japan; 2 Genetic Engineering and Biotechnology Department, Shahjalal University of Science and Technology, Sylhet 3114, Bangladesh; 3 Biochemistry and Molecular Biology Department, University of Dhaka, Dhaka 1000, Bangladesh; 4 Department of Anatomy, Ultrastructural Cell Biology, Faculty of Medicine, University of Miyazaki, 5200 Kihara, Kiyotake, Miyazaki 889-1692, Japan; 5 Biosafety & BSL3 Laboratory, ICDDR,B, Mahakhali, Dhaka 1212, Bangladesh; 6 Department of Microbiology, Graduate School of Medical and Dental Sciences, Kagoshima University, 8-35-1 Sakuragaoka, Kagoshima 890-8544, Japan; 7 Department of Bacteriology, Faculty of Medical Sciences, Kyushu University, 3-1-1 Maidashi, Higashi-ku, Fukuoka 812-8582, Japan; 8 Institute of Low Temperature Science, Hokkaido University, Kita 19, Nishi 8, Kita-ku, Sapporo 060-0819, Japan

## Abstract

Shiga toxin 2 (Stx2), one of the most important virulence factors of enterohaemorrhagic *Escherichia coli* (EHEC), is encoded by phages. These phages (Stx2 phages) are often called lambda-like. However, most Stx2 phages are short-tailed, thus belonging to the family *Podoviridae*, and the functions of many genes, especially those in the late region, are unknown. In this study, we performed a systematic genetic and morphological analysis of genes with unknown functions in Sp5, the Stx2 phage from EHEC O157:H7 strain Sakai. We identified nine essential genes, which, together with the terminase genes, determine Sp5 morphogenesis. Four of these genes most likely encoded portal, major capsid, scaffolding and tail fiber proteins. Although exact roles/functions of the other five genes are unknown, one was involved in head formation and four were required for tail formation. One of the four tail genes encoded an unusually large protein of 2,793 amino-acid residues. Two genes that are likely required to maintain the lysogenic state were also identified. Because the late regions of Stx2 phages from various origins are highly conserved, the present study provides an important basis for better understanding the biology of this unique and medically important group of bacteriophages.

Bacteriophages (phages) are the most abundant living entities on earth. Some bacteriophages, such as lambda, P2, and T-numbered phages, have contributed to advances in genetics and molecular biology as model organisms. In principle, phages have been classified based on their morphology and are sometimes further subgrouped into families based on sequence homologies to prototype phages, such as lambda-like and P2-like phage families. Based on the recent advances in sequencing technologies, the number of sequenced phage genomes has been exponentially increasing, and a huge number of prophages integrated in bacterial genomes have also been identified. The data from these sequencing efforts have unveiled a surprisingly high level of genetic diversity among bacteriophages. Studies have also shown that bacteriophages, particularly those that can be integrated into the host chromosomes (called temperate phages), play more critical roles than was previously recognized in the evolution of a wide range of bacteria by carrying various genes in host cells^1^,^2^,^3^. In addition, this expanding sequence information about phages/prophages has identified a huge number of phage-encoded genes with unknown functions^4^,^5^. However, limited efforts have been devoted to functional analyses of such newly identified genes, leaving their functions uncharacterized even in phages of medical or industrial importance. Among such cases are Shiga toxin 2 (Stx2)-transducing phages (Stx2 phage) of enterohemorrhagic *Escherichia coli* (EHEC).

EHEC strains cause a range of diseases, from mild diarrhea to severe, life-threating infections, such as hemorrhagic colitis and hemolytic uremic syndrome^6^,^7^. While multiple virulence factors are required for EHEC pathogenesis, the key virulence factors involved in the onset of severe diseases are Stxs^6^,^8^, highly potent cytotoxins that inhibit protein synthesis in mammalian host cells by depurinating a specific adenine residue of the 28S rRNA^9^. Stxs are classified into two subtypes: Stx1 and Stx2. There are several variants in each subtype, all of which are encoded by bacteriophages^10^,^11^,^12^. The Stx1 gene in *Shigella dysenteriae* is also thought to be encoded by a prophage remnant^13^. Although EHEC strains produce one or more such Stxs, it is thought that Stx2-producing strains cause more severe infections than Stx1-producing strains^14^,^15^. The EHEC strains of the O157 serotype, the most prevalent and widely studied EHEC serogroup, produce Stx1, Stx2a, and Stx2c, alone or in combination^16^,^17^. While a high level of Stx2a is produced upon phage induction, the level of Stx2c produced by EHEC O157 is generally very low, even after phage induction, and the expression of Stx1 production is iron-regulated and not linked to phage induction^18^,^19^,^20^,^21^,^22^.

Stx phages show remarkable variations in genome size, genome composition, morphology, and host specificity^23^,^24^,^25^,^26^,^27^ but are generally regarded as lambda-like phages because they contain many genes homologous to those of the archetypal bacteriophage lambda^11^,^28^,^29^,^30^,^31^. Among the sequenced Stx phages of O157 strains^11^,^18^,^26^,^28^,^29^,^30^, the Stx1 and Stx2c phages are highly similar to lambda phage and share many early and late genes with lambda phage. Consistent with this conservation, they have morphologies similar to that of lambda phage, which is constituted by a hexagonal head and a long flexible and non-contractile tail, a feature of the *Siphoviridae* family. However, Stx2a phages of O157 strains, such as Sp5 of strain Sakai^32^, 933W of strain EDL933^28^, E86654-Stx2 of E86654^33^, have short tails and are thus morphologically classified into the *Podoviridae* family. In fact, the Stx2a phages encode many genes that are absent in phage lambda. In particular, most genes in the late region, which are required for phage morphogenesis, are distinct not only from those in phage lambda and from those in other well-studied phages belonging to the *Podoviridae* family. Thus, the functions of these genes are largely unknown, except for a gene located just downstream of the *stx2* gene (z1466 in phage 933W, corresponding to ECs1207 of O157 strain Sakai), which was recently found to encode an esterase^34^. However, due to the difficulties in accurately enumerating plaque numbers and in obtaining high-titer phage lysates for Stx2a phages, conventional biochemical and genetic approaches to analyze the functions of each gene are not easily applicable. Although the former problem was partially solved by recent development of a sensitive and simple plaque formation method^35^, it is still difficult to perform biochemical analyses using purified phage particles, such as protein composition determination, becuase Stx2a phages yield only a low titer of phage lysate (up to 10^6^ PFU/ml). Therefore, a different approach needs to be employed to analyze the functions of Stx2a phage genes.

Here, we describe the results of a systematic genetic and morphological analysis of the genes of unknown function encoded by a representative Stx2a phage, Sp5 of O157:H7 strain Sakai^32^,^36^. We constructed a nearly complete set of in-frame deletion mutants of all Sp5 genes with unknown functions, systematically analyzed the excision, circularization, plaque formation, and lysogenization capacities of each mutant, and newly identified nine genes essential for infectious Sp5 phage particle formation. The results of electron microscopic examinations, including observation using an immunogold labeling technique, are also presented to assign the proteins’ functions in Sp5 morphogenesis.

## Results and Discussion

### Construction of a set of Sp5 mutants to determine the functions of unknown genes

**Figure 1:**
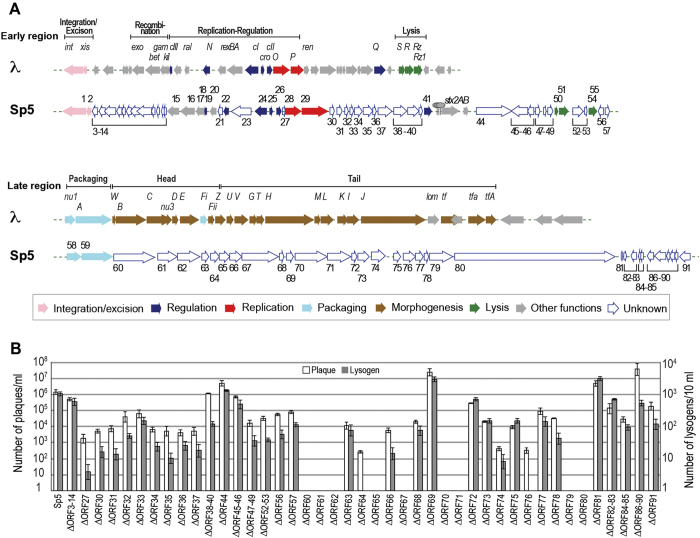
Genomic Structures of Sp5 and the plaque formation and lysogenization capacities of mutant Sp5 phages. (**A**) The genomic organizations of lambda phage and Sp5, the Stx2 phage of EHEC O157:H7 strain Sakai. Early and late regions are shown separately. Genes of known or reliably predicted functions^32^,^56^ are colored according to the functional category shown in the inset. The open arrows in Sp5 are the genes of unknown function that were analyzed in this study. ORFs deleted *en bloc* are indicated. (**B**) The plaque formation and lysogenization capacities of the WT and mutant Sp5 phages are shown. Error bars represent the standard deviations of three independent experiments.

The Sp5 genome is 62,708 bp long^36^. Among 91 protein-coding genes (open reading frames – ORFs) identified on the Sp5 genome (ECs1160 to ECs1251 in the O157 Sakai genome annotation; for convenience, these genes are referred to as ORF1 to ORF91 in this article; note that five small ORFs annotated in the original report of the genome sequence for O157 Sakai were excluded from the list of ORFs and four previously not-annotated ORFs were included; see Supplementary Table S1 for more details), the functions of 24 genes were reliably predicted based on high sequence homologies to functionally characterized genes from other phages, especially those from lambda phage^37^. These ORFs included genes for integration, replication, and gene regulation, and those encoding two terminase subunits (Fig. 1A and Supplementary Table S1). However, the remaining 67 ORFs lacked reliable database matches, so their functions were unpredictable. In particular, most genes in the late region were unique, and we were unable to confidently predict their functions^32^.

To characterize these genes with unknown functions, we constructed marker-free deletion mutants for these genes on the K-12 MG1655 genetic background. The parent phage used for mutant construction was an Sp5 derivative that was transferred to K-12 MG1655 from O157 Sakai, the *stx2* gene of which was replaced with the chloramphenicol resistance (Cp^r^) gene^32^. We used this Cp^r^-marked phage as the wild-type (WT) Sp5 throughout this study. As all deletions introduced were in-frame and marker-free deletions, polar effects were mostly avoidable. Several genes were deleted *en bloc*. Although we could not obtain deletion mutants of two ORFs in the early region, ORF21 (ECs1180) and ORF23 (ECs1184), we finally constructed 43 mutants and subjected them to the subsequent functional analyses (Fig. 1A).

The exact reason(s) for our failure to delete ORF21 and ORF23 are currently unknown, but the deletion of these genes appears to be lethal to the host organism. They are most likely required for the stable maintenance of the lysogenic state of Sp5. In the database search, both ORFs showed high homologies to many hypothetical proteins of prophages from EHEC and other bacteria. No conserved domains were found in these two ORFs.

### Excision and circularization of mutant phage genomes

We first determined whether each mutant could be excised from the host chromosome and could form an extrachromosomal circular intermediate. For this purpose, K-12 MG1655 lysogens for each mutant were treated with mitomycin C (MMC), and their total cellular DNA was isolated. Then, circularized phage genomes were detected by PCR using a pair of primers that specifically amplified the attachment site (*attP*)-containing region of Sp5, which is created upon prophage excision (Supplementary Table S2). In this analysis, we detected a single PCR product of an expected size for all K-12 lysogens, indicating that all mutant phages could be excised from the host chromosome to form a circular intermediate (Supplementary Fig. S1).

### Plaque formation and lysogenization of mutant phages

To identify the essential genes of Sp5, plaque formation and lysogenization assays were performed for each of the mutant phages. For the plaque formation assay, phage lysates were prepared by MMC treatment of each lysogen, and the plaque-forming units in each lysate were determined using a modified double agar overlay method that we previously developed to easily and accurately enumerate plaques of Stx2a phages^35^. This analysis revealed that nine out of the 43 mutants examined formed no plaques (Fig. 1B).

For the lysogenization assay, phage lysates from each mutant Sp5 phage lysogen were incubated with K-12 MG1655 cells, and the efficiency of lysogenization was determined by counting Cp^r^ colonies. This assay revealed that all nine mutants that were deficient in plaque formation were also unable to form lysogens (Fig. 1B), confirming their inability to infect K-12 cells. We could not detect any lysogens from two additional mutants, ΔORF64 and ΔORF76, under standard conditions. However, several Cp^r^ colonies were obtained when concentrated lysates were used. Therefore, these two mutants retained the capacity to infect and propagate in K12, but their capacity to lysogenize was significantly reduced. The biological significance of this somewhat unexpected behavior of ΔORF64 and ΔORF76 is currently unknown, but these two genes may be required for the efficient infection and/or lysogenization of Sp5. In contrast, three mutants, ΔORF69, ΔORF81, and ΔORF86–90 produced several times more plaques and lysogens than the WT (only a higher plaque formation ability was detected for ΔORF86–90), suggesting that ORF69, ORF81, and some ORFs in the ORF86–90 gene cluster may have some regulatory functions.

These results indicate that nine ORFs (ORFs60, 61, 62, 65, 67, 70, 71, 79, and 80; all are located in the late region) are essential for Sp5 infectivity (Fig. 1 and Table 1). Therefore, our following analysis focused on these nine genes.

### Complementation analysis of the nine newly identified essential genes

To verify that the products of the nine genes are required for Sp5 infection and propagation, we examined whether the deficiencies in lysogenization of the nine mutants were each able to be complemented *in trans* with plasmid-expressed gene products. In this analysis, we employed lysogenization assays because, if relevant gene products are provided during the phage induction process in K-12 Sp5 lysogens, phage particles which package the Sp5 genome, inject them into recipient cells, and generate Sp5 lysogens, could be produced. In this complementation analysis, lysogenization assays should be more sensitive than plaque formation assays where each gene product needs to be efficiently supplied not only in the phage induction process but also in recipient cells (during the plaque formation process). In fact, when each mutant phage was induced in K-12 cells that contained a plasmid encoding a relevant cloned gene, all phage lysates yielded Sp5 lysogens in K-12 (Table 1). These results indicate that the nine genes encode proteins essential for producing infectious Sp5 phage particles and also that the constructed deletion mutants do not carry any other fortuitous mutations.

### Identification of genes required for genome packaging

**Figure 2:**
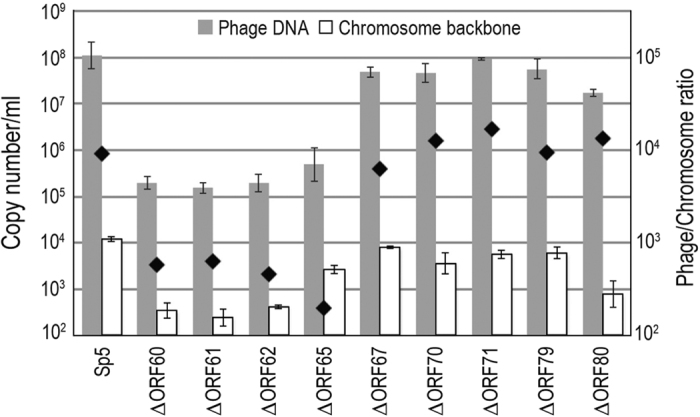
Quantification of packaged phage DNA in WT and mutant Sp5 phages. Packaged (and thus DNase-resistant) phage DNA was quantified via qPCR. For normalization, DNA preparations corresponding to those obtained from 1 ml of phage lysate were used as the template in all cases. The chromosomal DNA from a chromosome backbone region (CB) was quantified as a control. The average copy numbers of each genome are shown. Error bars represent the standard deviations of three independent experiments. The ratios of phage DNA and chromosomal DNA are indicated with black diamonds.

The process of phage particle formation for double-stranded (ds) DNA phages begins with head formation and packaging genomic DNA therein. To determine whether the nine mutant phages can package their genomes, we quantified their packaged (and thus resistant to DNase treatment) genomic DNA using quantitative PCR (qPCR), as described in the Methods. After MMC treatment, WT Sp5 yielded approximately 10^8^ copies of DNase-resistant genomes per one-milliliter culture (Fig. 2). Compared to the WT phages, remarkably lower copy numbers of genomic DNA were detected in the phage lysates from four mutants (ΔORF60, ΔORF61, ΔORF62, and ΔORF65), whereas those from five mutants (ΔORF67, ΔORF70, ΔORF71, ΔORF79, and ΔORF80) were compatible with those from the WT phages. In this assay, a low amount of chromosomal DNA was detected in all phage lysates examined (open bars in Fig. 2), likely due to the incomplete digestion of unpackaged DNA, the non-specific packaging/incorporation of chromosomal DNA into phage particles or membrane vesicles. Therefore, the phage DNA detected in the DNase-treated lysates from the former four mutants are also likely generated by similar mechanisms. In fact, the ratios of phage DNA to chromosomal DNA in the lysates from the four mutants were 100–1,000 times lower than that from the WT phage, while those from the latter five mutants were in the same range as the WT (indicated by diamonds in Fig. 2).

These results indicate that the mutants of ORF60, ORF61, ORF62, and ORF65 are deficient in genomic DNA packaging; therefore, these genes are required for the head formation and/or genome packaging processes. The results also indicate that head formation and genome packaging proceed properly in the mutants of ORF67, ORF70, ORF71, ORF79, and ORF80; thus, these five genes are required for tail formation (Table 1).

### Bioinformatics and morphological analyses of the nine essential genes

The first step in the head formation of dsDNA phages is the assembly of an intermediate called the prohead or procapsid. The prohead is a DNA-free head structure into which phage DNA is subsequently packaged. The prohead mainly consists of the portal protein, the major head protein, and the scaffolding protein, and its assembly process is well conserved among dsDNA phages and starts with the copolymerization of scaffolding proteins and major capsid proteins^38^, while, in HK97 and T5, the N-terminal domain of their major head proteins may perform a scaffolding function^39^,^40^. Coupled with genomic DNA packaging through the portal protein by the action of the terminase complex, scaffolding proteins are degraded by prohead proteases (e.g., λ and T4) or leave the procapsid without being degraded (e.g., P22 and φ29), providing the space for genomic DNA^38^,^41^. After DNA packaging, the terminase complex leaves the portal vertex, and head completion protein(s) attach there instead. Although the tail and head are formed separately and are finally joined together in phages of the *Siphoviridae* and *Myoviridae* families^38^,^41^, the tails of the *Podoviridae* family phages are formed on the portal vertex^42^.

**Figure 3:**
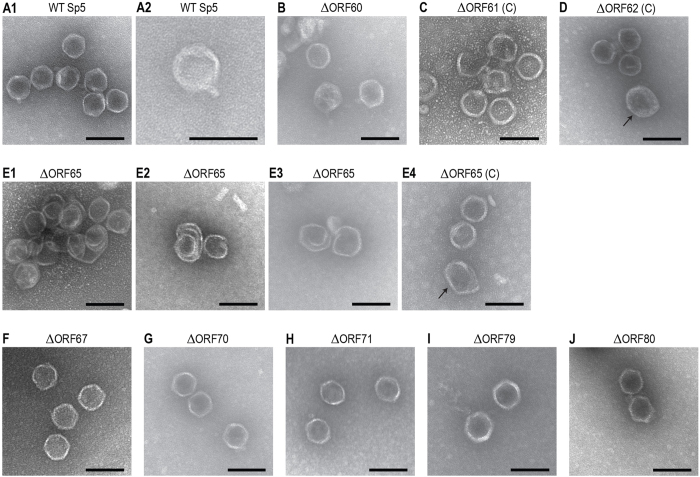
Electron microscopic analysis of the negative stained WT and mutant Sp5 phages. (**A**) WT Sp5. Short tail-like structures were occasionally observed, as shown in panel A2. (**B–J**) Sp5 mutants. Mutants complemented with the plasmid-encoded cloned genes (indicated by (**C**) added after mutant names) are also shown when clear morphological differences were observed compared to the non-complemented mutants. Refer to the text for detailed descriptions of the morphologies of each mutant. An arrow in panel D indicates an outer membrane vesicle-like particle. Some amounts of aberrant phage heads were observed in every sample from complemented head mutants as indicated by an arrow in panel E4. Electron micrographs were taken at magnifications of 60 K using a transmission electron microscope and were negatively contrasted with 2% uranyl acetate dihydrate. Bar, 100 nm.

**Figure 4:**
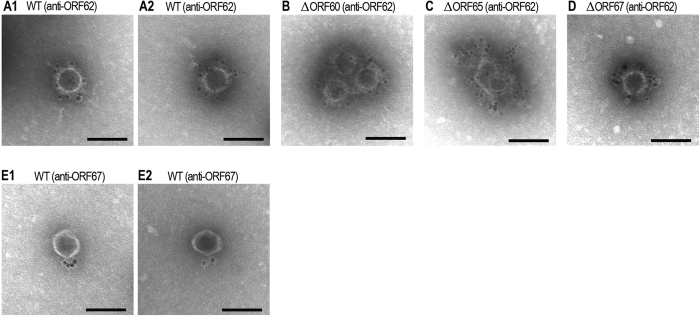
Immunoelectron microscopic analysis of the WT and mutant Sp5 phages. Immunoelectron microscopic analyses were performed using the anti-ORF62 (**A–D**) or anti-ORF67 antibodies (**E**). WT and mutant Sp5 phages were initially treated with primary antibodies and then treated with secondary antibody-conjugated gold particles. Bar, 100 nm.

To explore the functions/roles of the nine newly identified essential genes of Sp5 in its morphogenesis, we re-examined the conserved domains in each gene product and their sequence homologies to known proteins. Then, we performed electron microscopic (EM) analyses of the mutants of each gene and those complemented with plasmid-cloned genes (Fig. 3). In addition, we conducted immune-EM examinations via the immuno-gold labeling technique using antibodies specific to three gene products (ORF61, ORF62, and ORF67) (Fig. 4). We generated recombinant genes fused to the glutathione S-transferase (GST) gene for each of the nine genes to obtain purified recombinant proteins that could be used for specific antibody production. However, we were only able to obtain specific antibodies against three ORFs due to the high insolubility of other recombinant proteins. In this section, we first describe the characterization of ORF62 and ORF67 and then that of the other ORFs because ORF62 and ORF67 were confidently identified as the major capsid protein and the tail fiber protein, respectively, and their antibodies can be useful probes to detect head (or head-like) and tail structures.

ORF62 (ECs1223): A conserved domain search indicated that ORF62 belongs to the P22 coat protein superfamily and contains the DUF4043 and capsid_maj_N4 domains. However, ORF62 exhibited very low or no significant homology to the P22 coat (Gp5) and N4 (Gp56) proteins, while it showed high sequence similarities (up to 85% sequence identity) to hypothetical or “putative virion structural” proteins of many uncharacterized phages. In the EM analysis of ΔORF62, although a large number of outer membrane vesicle (OMV)-like particles were observed, no phage-head like particles were detected (data not shown). By complementation, heads with regular hexagonal outlines and of approximately 64 nm in diameter, which were similar to the WT (Fig. 3A), were observed (Fig. 3D), indicating that normal head formation was restored. The anti-ORF62 antibody was bound to the whole surface of the WT Sp5 head (Fig. 4A), but this binding was abolished in the ΔORF62 mutant (data not shown). These findings indicate that ORF62 is most likely the major capsid protein of Sp5.

ORF67 (ECs1228): Although a close homologue of ORF67 in phage 933W (L0121) was annotated as “tail-fiber-like”^28^, ORF67 exhibited no significant homologies to characterized phage proteins. However, it contains the phage tail fiber C-terminus domain and the phage tail fiber repeat 2 (Table 1). Multiple collagen-like domains with the repeating collagen signature sequence (Gly-X-Y)*n* are also present in ORF67, as in the tail fiber proteins from several phages^43^. In the EM examination of ΔORF67, apparently intact phage head structures were observed (Figs 3F and 4D). In the immuno-EM analysis of the WT phage using the anti-ORF67 antibody, multiple gold particles were bound to the tail ends of the phage particles (Fig. 4E). The binding of the anti-ORF67 antibody was not observed for the ΔORF67 mutant (data not shown). These observations indicate that ORF67 msot likely encodes the tail fiber protein of Sp5, which probably recognizes the YaeT (BamA) protein, a proposed *E. coli* surface receptor for Sp5^35^ and other short-tailed Stx2 phages^44^.

**Figure 5:**
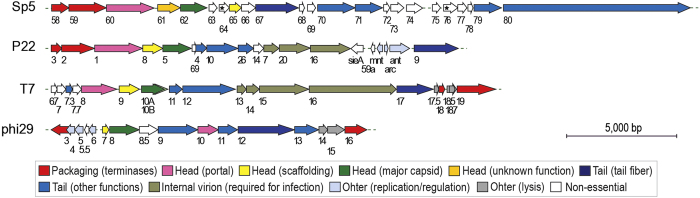
Comparison of the Sp5 late region with three well-characterized *Podoviridae* family phages. The genes (or ORFs) of each phage are numbered according to Asadulghani *et al*.^32^ for Sp5, Pedulla *et al*.^4^ for P22, Dunn & Studier^68^ for T7, and accession number NC_008720 for phi29. Essential genes are colored, and open arrows indicate non-essential genes. Genes with the same or similar functions are depicted in the same color, as indicated in the inset. Head completion/neck proteins are included in the “tail formation” category. Note that the ORF64 and ORF76 mutants (marked by asterisks) showed markedly reduced infectivity but were still infectious (see Fig. 1 and the main text). In T7, gp10B (light green in the figure) encodes a minor capsid protein.

ORF60 (ECs1221): No conserved domains were found in ORF60. Although it showed remarkable sequence similarities (up to 79% identity) to proteins of many uncharacterized bacteriophages from various Gram-negative bacteria, all were annotated as hypothetical or “putative portal” proteins. In the EM analysis of ΔORF60, in addition to many OMV-like particles, a significant number of apparently normal head-like structures were observed (Fig. 3B). Although clear increase in the proportion of normal head-like structures was not observed by complementation (data not shown), the anti-ORF62 antibody was bound to the surfaces of these head-like structures (Fig. 4B). This finding suggests that the ΔORF60 mutant can produce a head-like structure that is difficult to distinguish from the normal head of the WT. As no binding of the anti-ORF67 antibody was detected for the ΔORF60 mutant (data not shown), tails were likely not formed on these head-like structures. As mentioned above, this mutant is defective in genomic DNA packaging (Fig. 2). Several phages, such as the lambda, P22, and phi29 phages, still produce apparently normal head-like structures (prohead or procapsid) in the absence of portal protein^41^,^45^,^46^,^47^, so the biological and morphological features of ΔORF60 indicate that ORF60 most likely encodes a portal protein of Sp5. This conclusion is further supported by the genomic location of ORF60; it is located just downstream of the terminase genes, like in many phages (Fig. 5).

ORF61 (ECs1222): No significant homology to known characterized proteins and no conserved domains were found for ORF61. In the EM analysis of ΔORF61, no phage-head-like particles were detected (data not shown), and normal head structures were formed by complementation (Fig. 3C). These results indicate that ORF61 is required for phage head formation. However, the anti-ORF61 antibody did not bind to either WT phage particles or any of the mutants constructed in this study (data not shown). Thus, we cannot assign a precise role to ORF61 in head formation.

ORF65 (ECs1226): Bioinformatics analysis provided no information to aid in the functional assignment of ORF65. The EM analysis showed that the ΔORF65 mutant produced a large number of highly deformed particles, which often showed spiral structures (Figs. 3E1, 2, 3E3). A significant number of normal head-like structures were produced by complementation (Fig. 3E4). The anti-ORF62 antibody was bound almost evenly to the spiral structures (Fig. 4C), indicating that these structures are made of ORF62, the major capsid protein. Such open spiral structures were observed for the scaffolding protein-less mutants of phages lambda, P22, and phi29^48^,^49^,^50^,^51^. Binding of the anti-ORF67 antibody to the ΔORF65 mutant was not detected (data not shown). These findings indicate that ORF65 is required for head formation and may play a scaffolding function.

ORF70 (ECs1232), ORF71 (ECs1233), and ORF79 (ECs1241): No information to aid in the functional assignments of these three ORFs was obtained from the bioinformatics analyses. ORF71 contains DUF1983 and COG4733 domains. Although both domains have been found in various bacteriophage tail component proteins, including the host specificity protein (gpJ) of lambda phage^52^, their functions are unknown. In the EM examination of their mutants, head structures indistinguishable from that of the WT were observed (Fig. 3G–I). No binding of the anti-ORF67 antibody was detected for any of the three mutants. These results and their intact capacities to package phage DNA (Fig. 2) indicate that these ORFs participate in the Sp5 tail assembly process although their exact roles/functions are unknown.

ORF80 (ECs1242): This ORF encodes a very large protein consisting of 2,793 amino acid residues. ORF80 also showed no significant homologies to known characterized proteins, but contains the COG1483 (predicted ATPase, AAA+ superfamily) domain. The ΔORF80 mutant produced head structures indistinguishable from that of the WT phage (Fig. 3J), and binding of the anti-ORF67 antibody was not detected (data not shown). Thus, ORF80 participates in the Sp5 tail assembly process. Like ORF70, ORF71, and ORF79, the exact role/function of this extremely large ORF is unknown. However, the presence of the COG1483 domain suggests that ORF80 may be involved in some energy-requiring step in tail formation.

To briefly summarize this series of analyses, we identified the genes which most likely encode the portal (ORF60), major capsid (ORF62), scaffolding (ORF65), and tail fiber (ORF67) proteins of Sp5. Although the exact roles/functions of the other five essential genes are unknown, ORF61 is involved in head formation, and ORF70, ORF71, ORF79, and ORF80 are required for tail formation. The apparent absence of a protease indicates that, during the head formation process, the scaffolding protein of Sp5 leave the proheads without degradation, as has been shown for phages P22 and φ29^53^,^54^. The Sp5 gene product that corresponds to head completion proteins was not identified in this study.

### Organization of the Sp5 genes essential for its morphogenesis and comparison with other well-characterized *Podoviridae* family phages

Through a systematic analysis of Sp5 genes of unknown function, we identified nine essential genes required for its morphogenesis. Together with ORF58 and ORF59, which, respectively encode the small and large terminase subunits, these genes determine the essential processes of Sp5 morphogenesis. The number of essential late genes (11 ORFs) in Sp5 are roughly comparable to those in other well-characterized phages of the *Podoviridae* family: P22, T7, and phi29 (Fig. 5), but much smaller than the members in the *Siphoviridae* and *Myoviridae* families (21 and 18 genes in lambda and T5 belonging to *Siphoviridae*, respectively; 24 and 49 genes in P2 and T4 belonging to *Myoviridae*, respectively). However, the Sp5 late region is markedly longer than those of other phages. This difference is mainly due to the presence of an extremely large ORF (ORF80) but is also partly due to a significantly larger number of non-essential genes that are dispersed in the late region. Among these genes, ORF68 encodes a small polypeptide of 89 amino acids that comprise an apparent phage fiber C-terminus domain^55^,^56^. Although we found no sequences that can potentially induce a programmed frame shift to produce a tail fiber with an altered C-terminal sequence by fusing ORF67 and ORF68, ORF68 may represent an intragenomic reservoir sequence to create a variation in the tail fiber protein. The functions of ORF64 and ORF76 are also interesting. Both genes may be required for the efficient infection and/or lysogenization of Sp5 because their deletion mutants showed significantly reduced infection and lysogenization capacities (Fig. 1).

## Conclusion

Through systematic genetic and morphological analyses of the Sp5 genes of unknown function, we identified nine genes essential for its morphogenesis and two early genes that are likely required for the stable maintenance of the lysogenic state of Sp5. Among the nine essential late genes, we were able to assign the most likely functions to four genes, which encoded the portal, major capsid, scaffolding, and tail fiber proteins. The exact roles/functions of the other five genes are unknown, but one is required for head formation and the other four genes are required for tail formation. One of the four genes encodes an unusually large protein of 2,793 amino acid residues. These nine genes, together with two genes encoding the large and small subunits of terminase, determine the essential processes for Sp5 morphogenesis. As many Stx2 phages from various EHEC strains (O157 and non-O157 EHECs) contain the late regions same or very similar to that of Sp5 and share the 11 essential genes and most non-essential late genes, the present study is an important first step in understanding the biology of this unique and medically important, but not yet well-characterized, group of bacteriophages.

## Methods

### Bacterial strains, culture media and bacteriophages

The bacterial strains, bacteriophages, and plasmids used are listed in Table 2. Bacteria were grown under aerobic conditions at 37 °C in Lysogeny broth (LB) or on LB agar. When necessary, culture media or plates were supplemented with 40 μg/ml chloramphenicol (Cp), 100 μg/ml ampicillin (Ap), 25 μg/ml kanamycin (Km) or 100 μg/ml spectinomycin (Spec). *E. coli* K-12 MG1655 was used as the host for the Sp5 phage. A derivative of Sp5, in which *stx2* was replaced by the *cat* gene^32^, was used as the wild-type phage, and this derivative (Sp5*stx2::cat*) is referred to as the WT Sp5 in this article.

### Bioinformatics analysis of ORFs

Homology searches were performed using BLASTP through the National Center for Biotechnology Information (NCBI; http://www.ncbi.nlm.nih.gov/). The conserved domains and motifs were searched using Conserved Domain Database (CDD)^57^, InterProScan^58^, and SMART^59^.

### Mutant construction

To avoid the polar effects that may be caused by deletion and/or marker insertion, marker-free in-frame deletion mutants of Sp5 were constructed under the K-12 MG1655 background as described previously^60^, with some modifications. Flanking regions with nine nucleotides from each end of the target ORFs were amplified using the primers listed in Supplementary Table S2 and the genomic DNA of K-12 Sp5 lysogen as a template to obtain two amplicons. These two amplicons were combined together and used as the template to generate an in-frame deleted PCR product of the desired ORF by overlap extension PCR (OE-PCR) using adapter-PCR primers (Supplementary Table S2). The resulting PCR product was purified by gel extraction and cloned into pDONR201 (Gateway cloning system, Invitrogen) by a BP-reaction to obtain pDONR201-ΔORF#. The recombinant product was introduced into DH5α by transformation. Clones were selected on Km plates along with negative selection for the *ccdB* death cassette, which remains in pDONR201 if the expected recombination does not occur. In-frame deletion in each clone was confirmed by PCR using M13 primers and capillary sequencing. The ORF containing an in-frame deletion was cloned into the *sacB*-containing positive suicide vector pABB-CRS2^61^ by the LR recombination reaction (Gateway cloning system, Invitrogen) to obtain pABB-CSR2-ΔORF#. This construct was transformed into *E. coli* SM10 λpir and selected on Ap plates. The plasmid was transconjugated into an Sp5 lysogen of K-12 MG1655, and its resistance to Ap and Cp was confirmed on Ap and Cp plates. Integration of pABB-CSR2-ΔORF# by a single crossover was confirmed by PCR using primers listed in Supplementary Table S2. Confirmed transconjugants were grown without selection until the late logarithmic phase in LB at 37 °C to allow the excision of the *sacB*-containing vector and were then spread on LB agar plates containing 5% sucrose. Finally, the in-frame deletion in each sucrose-resistant and Ap-sensitive colony was confirmed by PCR using respective ORF primers (Supplementary Table S2) and capillary sequencing.

### Preparation of phage lysates

Phage lysates were prepared as described previously^32^, with a few modifications. Briefly, K-12 Sp5 lysogens were grown overnight at 37 °C in Cp-containing LB. The cell concentration was adjusted to an optical density at 600 nm (OD_600_) of 0.3–0.4 with LB. Diluted cultures were grown for 50–55 min at 37 °C, and MMC was added to a final concentration of 1.0 μg/ml. After 6–7 h of incubation, the culture was treated with a few drops of chloroform and centrifuged at 14,000xg for 50 min at 4 °C. The supernatant was filter-sterilized with Minisart 0.22-μm pore size filters (Sartorius) and incubated with 20% polyethylene glycol 8000/10% NaCl for phage precipitation. Precipitated phage particles were pelleted by centrifugation at 24,000x g for 1 h at 4 °C and were resuspended in SM buffer (0.58% NaCl, 0.2% MgSO_4_.7 H_2_O, 50 mM Tris–HCl (pH 7.5), and 0.01% gelatin) in a 1/100 volume of the original supernatant. The resuspended phage lysate was mixed with a 0.1 volume of chloroform and briefly centrifuged. The supernatant was collected and incubated at 37 °C for 20 min to remove the residual chloroform and was used for analyses as “phage lysate”.

### Plaque formation and lysogenization assays

Plaque formation and lysogenization assays were performed as described previously^35^. In the lysogenization assay, several colonies were randomly selected, and the lysogenization of Sp5 in these colonies was confirmed by colony PCR using primers Sp5_LysF and Sp5_LysR (Supplementary Table S2).

### Complementation assay

Complementation experiments were performed using pTB101, an expression vector containing the *tac* promoter and *lacI*^*q*^^62^. The entire coding region with the presumed Shine-Dalgarno (SD) sequence of the target ORF was PCR-amplified with a pair of primers, each containing EcoRI and SalI restriction sites (Supplementary Table S2). The amplicons were gel-purified, digested with the two enzymes and ligated into the EcoRI/SalI-double digested pTB101. After confirming the sequence of the cloned ORF by PCR and sequencing, the construct was transformed into K-12 containing the respective Sp5 mutants. The transformed cells were grown at 37 °C in LB containing Cp and Spec. The cloned ORF was induced with isopropyl-beta-D-1-thiogalactopyranoside (IPTG) at a final concentration of 0.025 mM 30 min after the MMC induction. After 6–7 h of incubation at 37 °C, the phage lysate was prepared and used for lysogenization assay and for EM analysis.

### Detection of excised phage genomes and quantification of DNase-resistant phage DNA in phage lysates

To detect the excised and circularized Sp5 genomes, qualitative PCR was performed using primers specific to each of the attachment site (*attP*)-flanking regions (Supplementary Table S2). The template DNA was prepared using a simplified alkaline DNA preparation method^63^. Briefly, after 1-h treatment with MMC, bacterial cells were collected by centrifugation at 8000x g for 5 min at 4 °C. The cell pellet was lysed with 25 mM NaOH for 10 min at 95 °C and then neutralized with 80 mM Tris-HCl. After a brief centrifugation, one microliter of the supernatant was used as a PCR template.

To quantify the DNase-resistant Sp5 DNA in phage lysates, each lysate prepared as described above was treated with DNase I (50 μg/ml) (Sigma-Aldrich) and RNase A (50 μg/ml) (Sigma-Aldrich) for 1 h at 37 °C. The sample was then treated with proteinase K (100 μg/ml; Wako Pure Chemical Industries, Ltd.) for 1 h at 50 °C, and the DNA was isolated via phenol:choloroform extraction and ethanol precipitation and quantified by qPCR using the StepOnePlus™ Real-Time PCR system and TaqMan Fast Advanced Master Mix (Applied Biosystems). The concentrations of the TaqMan probes and amplification primers used for qPCR were 250 nM and 400 nM, respectively. For TaqMan probes, FAM (5′) and TAMRA (3′) were used as the reporter and quencher dyes, respectively. Primer Express™ (PE BioSystems) was used to design TaqMan probes and primers (Supplementary Table S2). The qPCR was performed for 45 cycles at 95 °C for 15 sec and 60 °C for 1 min. Phage DNA isolated from 1 ml of culture was used as a template. Standard curves with a dynamic range from 10^2^ to 10^8^ copies/μl were generated for each amplicon. Residual chromosomal backbone DNA in DNase-treated phage lysates was also quantified as a control.

### Protein expression, purification and antibody preparation

Full-length target ORFs were PCR-amplified and cloned into the pGEX-6p-1 expression vector (GE Healthcare) to generate genes encoding GST-fusion proteins. The sequences of cloned ORFs were confirmed by DNA sequencing. The recombinant plasmids were introduced into *E. coli* BL21 (DE3) cells^64^,^65^, which were grown at 37 °C in Ap-containing LB. To induce protein expression, IPTG was added at a concentration of 0.1 mM when the OD_600_ reached 0.6, and the cells were then cultured for 3 h at 25 °C. The cells were harvested by centrifugation at 5000x g at 4 °C, resuspended in phosphate-buffered saline (PBS) (pH 7.3), lysed by sonication and then separated into soluble and insoluble fractions by centrifugation at 14,000x g at 4 °C. Proteins in each fraction were analyzed by sodium dodecyl sulfate polyacrylamide gel electrophoresis (SDS-PAGE). GST-fusion proteins in soluble fractions were affinity-purified using Glutathione Sepharose 4B (GE Healthcare) according to the manufacturer’s instructions, and the purities of those proteins were confirmed by SDS-PAGE. Rabbit antibodies against purified proteins were prepared by Sigma-Aldrich Japan. The titers and specificities of each antiserum were determined via enzyme-linked immunosorbent assays or Western blotting using horseradish peroxidase-conjugated goat anti-rabbit IgG antibodies (Bio-Rad).

### Electron microscopy and immunoelectron microscopy

A drop of phage suspension was loaded onto a formvar-carbon-coated copper grid and allowing it to sediment for 1 min at room temperature (RT). Excess liquid was removed with Whatman blotting paper, and the copper grids were then negatively contrasted with 2% uranyl acetate. Phage morphologies were analyzed using the HT7700 transmission EM (Hitachi) operating at 80 kV.

Monodisperse colloidal gold (CG) 8 nm in diameter was prepared as described previously^66^. The conjugation of CG particles to goat anti-rabbit IgG (Vector Laboratories) was performed as previously described^67^. For the immunoelectron microscopy analysis, a drop of phage suspension was placed onto a 150-mesh gold grid coated with Formvar film and incubated for 10 min at RT, and excess liquid was removed using Whatman blotting paper. The grids were incubated with 1% bovine serum albumin (BSA) and were incubated with primary antibodies (1:500) for 1 h at RT. After being rinsed with distilled water (DW), the grids were incubated with goat anti-rabbit IgG-CG conjugate (1/50 diluted with 1% BSA) for 40 min at RT. Each grid was then washed with DW and fixed with 1% glutaraldehyde-cacodylate-buffer (pH 7.4). The samples were negatively stained with 2% uranyl acetate and examined with Hitachi HT7700.

## Additional Information

**How to cite this article**: Mondal, S. I. *et al*. Genes essential for the morphogenesis of the Shiga toxin 2-transducing phage from *Escherichia coli* O157:H7. *Sci. Rep.* **6**, 39036; doi: 10.1038/srep39036 (2016).

**Publisher's note:** Springer Nature remains neutral with regard to jurisdictional claims in published maps and institutional affiliations.

## Supplementary Material

Supplementary Information

## Figures and Tables

**Table 1:** The essential genes identified in this study, the phenotypes of their deletion mutants, and the results of bioinformatics analysis of each gene.

ORFs (ECs)	Excision	Lysogen-ization	Plaque formation	Comple-mentation^a^	Packaging	Superfamily/Conserved domains
ORF21 (ECs1180)	NA	NA	NA	NA	NA	No
ORF23 (ECs1184)	NA	NA	NA	NA	NA	No
ORF60 (ECs1221)	+	−	−	+	−	No
ORF61 (ECs1222)	+	−	−	+	−	No
ORF62 (ECs1223)	+	−	−	+	−	P22 coat protein superfamily
ORF65 (ECs1226)	+	−	−	+	−	No
ORF67 (ECs1228)	+	−	−	+	+	phage tail fiber C-terminus domain, phage tail fiber repeat 2, multiple collagen like domains
ORF70 (ECs1232)	+	−	−	+	+	No
ORF71 (ECs1233)	+	−	−	+	+	No
ORF79 (ECs1241)	+	−	−	+	+	No
ORF80 (ECs1242)	+	−	−	+	+	predicted ATPase, AAA^+^ superfamily

^a^Complementation was examined in lysogenization assays.

NA, not applicable; these two ORFs are not deletable.

**Table 2:** Bacterial strains, phage, plasmids used in this study.

Strains, phage, and plasmids	Features	References or sources
Bacterial strains:		
* E. coli* MG1655	F^−^, lambda^−^, rph-1	69
* E. coli* BL21 (DE3)	F^−^, ompT, hsdS (r^B−^, m^B−^), gal	64,65
* E. coli* Sm10λpir	Permissive strain for replication of pABB-CSR2	61
Phage		
* *Sp5	*Δstx2::cat*-cassette	32
Plasmids:		
* *pKD4	FRT-flanked Tn5 neomycin phospho- transferase, Ap^r^, Km^r^	70
* *pKD46	Lambda red helper plasmid, Ap^r^	70
* *pDONR201	Gateway cloning vector	Invitrogen
* *pABB-CRS2	Suicide vector	60
* *pDONR-ORF#	pDONR201-derivatives containing a deletion target ORF (# indicates the ORF number)	This study
