# Supplementary material for: Genes essential for the morphogenesis of the Shiga toxin 2-transducing phage from Escherichia coli O157:H7
Source: Sci Rep. 2016 Dec 14;6:39036. doi: 10.1038/srep39036 (PMC5155283; doi:10.1038/srep39036)
Supplement: Supplementary Information [file srep39036-s1.pdf]

Supplementary data

Genes essential for the morphogenesis of the Shiga toxin 2-transducing phage from *Escherichia coli* O157:H7

Shakhinur Islam Mondal,[1,2] Md Rakibul Islam,[3] Akira Sawaguchi,[4] Md Asadulghani,[5] Tadasuke Ooka,[6] Yasuhiro Gotoh,[7] Yasuhiro Kasahara,[8] Yoshitoshi Ogura,[7] and Tetsuya Hayashi[7]

[1]Division of Microbiology, Department of Infectious Diseases, Faculty of Medicine, University of Miyazaki, 5200 Kihara, Kiyotake, Miyazaki 889-1692, Japan; [2]Genetic Engineering and Biotechnology Department, Shahjalal University of Science and Technology, Sylhet 3114, Bangladesh; [3]Biochemistry and Molecular Biology Department, University of Dhaka, Dhaka 1000, Bangladesh; [4]Department of Anatomy, Ultrastructural Cell Biology, Faculty of Medicine, University of Miyazaki, 5200 Kihara, Kiyotake, Miyazaki 889-1692, Japan; [5]Biosafety & BSL3 Laboratory, ICDDR,B, Dhaka 1212, Bangladesh; [6]Department of Microbiology, Graduate School of Medical and Dental Sciences, Kagoshima University, 8-35-1 Sakuragaoka, Kagoshima 890-8544, Japan; [7]Department of Bacteriology, Faculty of Medical Sciences, Kyushu University, 3-1-1 Maidashi, Higashi-ku, Fukuoka 812-8582, Japan. [8]Institute of Low Temperature Science, Hokkaido University, Kita 19, Nishi 8, Kita-ku, Sapporo 060-0819, Japan

Email: thayash@bact.med.kyushu-u.ac.jp

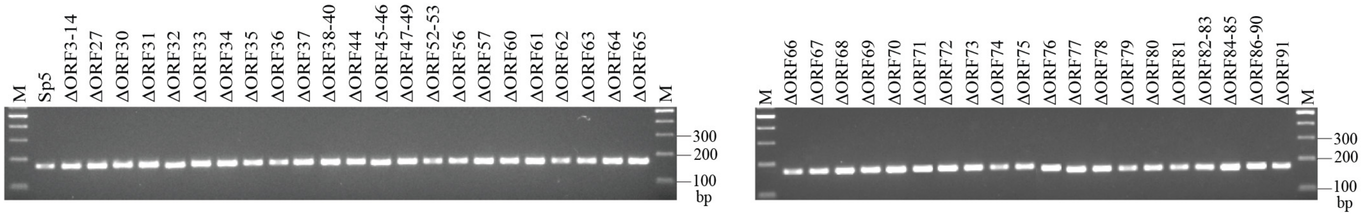

Supplementary Fig S1: Excision and circularization of WT and mutant Sp5 genomes. The results of an agarose gel electrophoretic analysis of the PCR products derived from the excised and circularized genomes of the WT Sp5 and all mutants generated in this study are shown.

Supplementary Table S1. List of the ORFs on the Sp5 genome

ORF (ECs)	Gene products	Length (AAs)
ORF1 (ECs1160)	Integrase	436
ORF2 (ECs1161)	Excisionase	94
**ORF3 (ECs1162)**	Hypothetical	103
**ORF4 (ECs1163)**	Hypothetical	114
**ORF5 (ECs1164)**	Hypothetical	207
**ORF6 (ECs1165)**	Hypothetical	95
**ORF7 (ECs1166)**	Hypothetical	72
**ORF8 (ECs1167)**	Hypothetical	95
**ORF9 (ECs1168)**	Hypothetical	155
**ORF10 (ECs1169)**	Hypothetical	257
**ORF11 (ECs1170)**	Hypothetical	73
**ORF12 (ECs1171)**	Hypothetical	71
**ORF13 (ECs1172)**	Hypothetical	63
**ORF14 (ECs1173)**	Hypothetical	60
ORF15 (ECs1174)	Exonuclease Exo	226
ORF16 (ECs1175)	Recombination protein Bet	261
ORF17 (ECs1176)	Nuclease inhibitor Gam	138
ORF18 (ECs1177)	Host killing protein Kil	89
ORF19 (ECs1178)	Repressor CIII	54
ORF20 (ECs1179)	ssDNA binding protein Ea10	122
**ORF21 (ECs1180)**	Hypothetical	83
ORF22 (ECs1181)	Antiterminator protein N	90
**ORF23 (ECs1184)**	Hypothetical	173
ORF24 (ECs1185)	Repressor CI	217
ORF25 (ECs1186)	Repressor protein	71
ORF26 (ECs1187)	Transcriptional activator CII	98
**ORF27 (ECs1188)**	Hypothetical	48
ORF28 (ECs1189)	Replication protein O	299
ORF29 (ECs1190)	Replication protein P	483
**ORF30 (ECs1191)**	Hypothetical	89
**ORF31 (ECs1192)**	Hypothetical	92
**ORF32 (ECs1193)**	Hypothetical	71
**ORF33 (ECs1194)**	Hypothetical	78
**ORF34 (ECs1195)**	Similar to the recombination protein NinB of phage lambda	148
**ORF35 (ECs1196)**	Similar to DNA methylases (Phage N-6-adenine-methyltransferases)	175
**ORF36 (ECs1197)**	Nearly similar to NinE of phage lambda	60
**ORF37 (ECs1199)**	Similar to the antirepressor protein of phage P22	244
**ORF38 (ECs1200)**	Putative DNA binding protein	240
**ORF39 (ECs1201)**	Similar to the recombination protein NinG of phage lambda	201
**ORF40 (ECs1202)**	Hypothetical	64
ORF41 (ECs1203)	Antiterminator protein Q	144
ORF42 (ECs1205)	Stx2A	319
ORF43 (ECs1206)	Stx2B	89
**ORF44 (ECs1207)**	Hypothetical	634
**ORF45 (ECs1208)**	Hypothetical	296
**ORF46 (ECs1209)**	Hypothetical	108
**ORF47 (ECs5606)**	Hypothetical	89
**ORF48 (ECs1210)**	Hypothetical	59
**ORF49 (ECs1211)**	Hypothetical	148
ORF50 (ECs1212)	Holin (for cell lysis)	71
ORF51 (ECs1213)	Endolysin (for cell lysis)	177
**ORF52 (ECs1214)**	Similar to the antirepressor protein of phage P22	189
**ORF53 (ECs5607)**	Hypothetical	44
ORF54 (ECs1215)	Endopeptidase Rz (for cell lysis)	154

ORF55 (ECs1216)	Lipoprotein precursor Rz1(for cell lysis)	61
**ORF56 (ECs1217)**	Nearly similar to the lambda Bor protein	97
**ORF57 (ECs1218)**	Hypothetical	77
ORF58 (ECs1219)	Terminase, small subunit	268
ORF59 (ECs1220)	Terminase, large subunit	568
**ORF60 (ECs1221)**	Similar to many hypthetical or putative portal proteins of uncharacterized phages	714
**ORF61 (ECs1222)**	Hypothetical	335
**ORF62 (ECs1223)**	Hypothetical	404
**ORF63 (ECs1224)**	Hypothetical	129
**ORF64 (ECs1225)**	Hypothetical	153
**ORF65 (ECs1226)**	Hypothetical	187
**ORF66 (ECs1227)**	Hypothetical	216
**ORF67 (ECs1228)**	Hypothetical	645
**ORF68 (ECs1229)**	Hypothetical	89
**ORF69 (ECs1230)**	Hypothetical	80
**ORF70 (ECs1232)**	Hypothetical	567
**ORF71 (ECs1233)**	Hypothetical	422
**ORF72 (ECs1234)**	Hypothetical	92
**ORF73 (ECs1235)**	Hypothetical	205
**ORF74 (ECs1236)**	Weakly similar to the Lom outer membrane protein of phage lambda	244
**ORF75 (ECs1237)**	Hypothetical	133
**ORF76 (ECs1238)**	Hypothetical	218
**ORF77 (ECs1239)**	Hypothetical	148
**ORF78 (ECs1240)**	Hypothetical	83
**ORF79 (ECs1241)**	Hypothetical	421
**ORF80 (ECs1242)**	Hypothetical	2793
**ORF81 (ECs5608)**	Hypothetical	31
**ORF82 (ECs1243)**	Hypothetical	61
**ORF83 (ECs1244)**	Hypothetical	114
**ORF84 (ECs5683)**	Hypothetical	51
**ORF85 (ECs1245)**	Hypothetical	70
**ORF86 (ECs1246)**	Hypothetical	131
**ORF87 (ECs1247)**	Hypothetical	219
**ORF88 (ECs1248)**	Hypothetical	72
**ORF89 (ECs1249)**	Hypothetical	94
**ORF90 (ECs1250)**	Hypothetical	73
**ORF91 (ECs1251)**	Similar to antirepressor proteins	209

“Note: Genes selected for mutant construction are bolded.
Group of genes that were deleted in block are indicated by boxes.
In this analysis, five small ORFs (ECs1182, 1183, 1198, 1204, and 1231) annotated in the original report of the genome sequence for O157 Sakai were excluded from the analysis as they seem unlikely to encode functional polypeptides. Instead, we included four ORFs (ECs5606, 5607, 5608, and 5683), which were previously not annotated but found to have some possibility to encode functional polypeptides, in the analysis. The positions of the four ORFs on the O157 Sakai genome (accession no. NC_002695) are 1271815-1272084 (ECs5606), 1274393-1274527 (ECs5607), 1304526-1304621 (ECs5608), and 1305418-1305573 (ECs5683)”.

Supplementary Table S2. Primers used in this study

**Primers/Probes**	**Sequences (5′–3′)**
**For analysing excision and circularization of phage genome**	
Sp5_CirF	CTCTTGGACGATCTTCGGTAA
Sp5_CirR	CGTGACTCAAGTTGCCATGT
**For confiming Sp5 lysogeny**	
Sp5_LysF	CGCGAAAGGCTCCATGAATG
Sp5_LysR	GCTGTTTCGTCTGACCTCCA
**For quantifying the Sp5 and chromosome DNA**	
Sp5DNA_F	CTCTTGGACGATCTTCGGTAA
Sp5DNA_R	CGTGACTCAAGTTGCCATGT
[a]CB_F	AGCACCAAAGAAGGCGTTCA
[a]CB_R	GGCCAATGCCAGATTTATTCA
**TaqMan Probes for DNA quantification**	
Sp5DNA_probe	CTTCATGGTTTCAACATGTCTAAGGAT
[a]CB	ATATCCTGGAAATCCTGCTCAACCT
[b]**For producing recombinant proteins**	
60_EcoR1F	GGC**GAATTC**AAAAATGAAACTAACACC
60_SalIR	GGC**GTCGAC**TCAGAGCGACATTTCATTCAT
61_EcoR1F	GGC**GAATTC**GATTTTGAATTTACGGGT
61_SalIR	GGC**GTCGAC**TTACCCGAGCTTCTCCAGAAG
62_BamF	GGC**GGATCC**ACGACTGTAACATCAGCC
62_SalIR	GGC**GTCGAC**TCAGAGCTTCACTGCTGTATC
65_EcoR1F	GGC**GAATTC**GCGGAACTGAGTGATTTT
65_SalIR	GGC**GTCGAC**TCAGAAAAACCTCTGCCTGCG
67_EcoR1F	GGC**GAATTC**AGTGTTGTTGTTTCGGGG
67_SalIR	GGC**GTCGAC**TCATTCTCCTGTTCTGCCTGT
70_SalIF	GGC**GTCGAC**TCATGCCGATCGCGATTTTA
70_Xho1R	GGC**CTCGAG**TCATACCGGCATCTCCGACAT
71_EcoR1F	GGC**GAATTC**ACCAGAAAACCGTGGCGT
71_Xho1R	GGC**CTCGAG**TCAGCGTAATCTGCCTATGCG
79_EcoR1F	GGC**GAATTC**AGCGGATTTGCACAGGGG
79_SalIR	GGC**GTCGAC**TTACTGGGCGTAATTCTGTTT
42a_BamF	GGC**GGATCC**GCCTATTCAGAGGAACAG
42a_SalR	GGC**GTCGAC**TTATGCGTAGTCCGGTTTCGG
42b_BamF	GGC**GGATCC**ATGTTCGTATGCCTCAGC
42b_SalR	GGC**GTCGAC**TTAAGCGGCAAATCCGGTGCA
42c_BamF	GGC**GGATCC**GCGGCGGCACACTATGAG
42c_SalR	GGC**GTCGAC**TTA CTGGAATTTATCCTGCAT
42d_BamF	GGC**GGATCC**AAGGGGCCGTATTACTCC
42d_SalR	GGC**GTCGAC**TTATTAATCCACTCCCACTTTTTT
42e_BamF	GGC**GGATCC**ACCGGCTGGTTTAACATG
42e_SalR	GGC**GTCGAC**TTATTAATCCACTCCCACTTTTTT
42f_Bam	GGC**GGATCC**AAGGGGCCGTATTACTCC
42f_SalR	GGC**GTCGAC**TTAAAGACGCAGAGCGTTTTC

For consructing deletion mutants	
ORF3-14(ECs1162-1173)_B1F	AAAAAGCAGGCTTGGGTACGGTTCTTCCTTTTC
ORF3-14(ECs1162-1173)_B1R	GCAATGGCGATGAAGCATCGCATCGAATAAGACGTAAC
ORF3-14(ECs1162-1173)_B2F	ATGCTTCATCGCCATTGC
ORF3-14(ECs1162-1173)_B2R	AGAAAGCTGGGTCTCCGGTTAACGATGAAACC
ORF3-14(ECs1162-1173)_ckF	TCTCCAGATACCCGTTTTCG
ORF3-14(ECs1162-1173)_ckR	ACGGCATAAAGCCATGATTC
ORF3-14(ECs1162-1173)_Seq	CTGGCGCGTAATATTGTTCC
ORF27(ECs1188)_B1F	AAAAAGCAGGCTCTGCGTCATACCTAGCTCTTC
ORF27(ECs1188)_B1R	GATTAGATATGTTACTCATGACCTTTTGTCATAATTACTCCTG
ORF27(ECs1188)_B2F	GGTCATGAGTAACATATCTAATC
ORF27(ECs1188)_B2R	AGAAAGCTGGGTCACCCTCACGAACTTTCAGG
ORF27(ECs1188)_ckF	AAGCCAGAATGCAGAGTCAC
ORF27(ECs1188)_ckR	TAAGCAAAACCGCCAACAGAC
ORF27(ECs1188)_Seq	GATTGGCTGTCATGAATCG
ORF30(ECs1191)_B1F	AAAAAGCAGGCTACAACGAGGAATGAGCACAG
ORF30(ECs1191)_B1R	GTGATGTAGATGGTCATTGCTTTTTATTCATCAGAATCCTCC
ORF30(ECs1191)_B2F	AAGCAATGACCATCTACATCAC
ORF30(ECs1191)_B2R	AGAAAGCTGGGTTCTCCGGTTCCATTTTTCAG
ORF30(ECs1191)_ckF	CTATGCAATGCAGGTTCGTG
ORF30(ECs1191)_ckR	GACTTCGTGGGGTGACTTGT
ORF30(ECs1191)_Seq	AACGCCCACTACCAAGTGAC
ORF31(ECs1192)_B1F	AAAAAGCAGGCTCCTGCTTTACATCGACGACA
ORF31(ECs1192)_B1R	GTGATATTTGCTTAATCGAAGAGAGGGAAGTGAACGA
ORF31(ECs1192)_B2F	TTCGATTAAGCAAATATCAC
ORF31(ECs1192)_B2R	AGAAAGCTGGGTCTCAGTCATGCTGGCCTTC
ORF31(ECs1192)_ckF	GAAATGCCAGGTGATCAGATACTG
ORF31(ECs1192)_ckR	GCCCTACCTTTTCGTTGTGA
ORF31(ECs1192)_Seq	ATTACGCGCCAGATGGTAAC
ORF32(ECs1193)_B1F	AAAAAGCAGGCTCACGGTGTATTGCATTCAGG
ORF32(ECs1193)_B1R	GCTCACTCCTTCACTTTAACGTACTCATTTCTTAAAACC
ORF32(ECs1193)_B2F	TTAAAGTGAAGGAGTGAGC
ORF32(ECs1193)_B2R	AGAAAGCTGGGTCGGTGGGTTATTCCAGATTG
ORF32(ECs1193)_ckF	GAACCTTGCCAAAGAGCTTG
ORF32(ECs1193)_ckR	TGCGAACTTCGTCAACACTC
ORF32(ECs1193)_Seq	AGAAGGTGCTTATGCGATGG
ORF33(ECs1194)_B1F	AAAAAGCAGGCTCGGATTCCTGAACGAAATTG
ORF33(ECs1194)_B1R	CGTCTATGGCGTTATTTCTGGTCGCTCATGCTCACTCCTTC
ORF33(ECs1194)_B2F	CAGAAATAACGCCATAGACG
ORF33(ECs1194)_B2R	AGAAAGCTGGGTTTCGTCGAGGACTGATGAAC
ORF33(ECs1194)_ckF	GGCACTACCAATAATCCAG
ORF33(ECs1194)_ckR	CAGGTGAGCCGTGTAATTGA
ORF33(ECs1194)_Seq	AGCTGAGCTTGTCAGGGCTA
ORF34(ECs1195)_B1F	AAAAAGCAGGCTTATTTGGGGGAGAGGGAAGT
ORF34(ECs1195)_B1R	GATTTGATAGTCATGCAGCTTGCTTCATCTCCTGCTCTC
ORF34(ECs1195)_B2F	GCTGCATGACTATCAAATC
ORF34(ECs1195)_B2R	AGAAAGCTGGGTCACAGCCCACCATAATTCAG
ORF34(ECs1195)_ckF	GCATGGGATGCACAAATATC
ORF34(ECs1195)_ckR	CGGGGAGAGAGTTTCAACAG
ORF34(ECs1195)_Seq	CAATGAGTTTCAGCGCAACA
ORF35(ECs1196)_B1F	AAAAAGCAGGCTGTACGATAGCTGAGCTTGTCAG
ORF35(ECs1196)_B1R	GCTGTCGCCTCATGCCGCGATAGTCATGCAGCCCTAC

ORF35(ECs1196)_B2F	GCGGCATGAGGCGACAGC
ORF35(ECs1196)_B2R	AGAAAGCTGGGTTCTCGCACTGGCTGAAATAGC
ORF35(ECs1196)_ckF	AGAAGGTGCTTATGCGATGG
ORF35(ECs1196)_ckR	AATCCCACAGCCATACAAGG
ORF35(ECs1196)_Seq	CGGAAGACTGGAAAGACCTG
ORF36(ECs1197)_B1F	AAAAAGCAGGCTACAAGTCACCCCACGAAGTC
ORF36(ECs1197)_B1R	CATCGCGTCAATCACCTTGTTCGCCTCATGCCGCCCTC
ORF36(ECs1197)_B2F	ACAAGGTGATTGACGCGATG
ORF36(ECs1197)_B2R	AGAAAGCTGGGTCCCTCCCGATGATATACGAG
ORF36(ECs1197)_ckF	TAATGCTCGTCTGTGGGTTG
ORF36(ECs1197)_ckR	CCGAAGCTGGGTTTGTTAAG
ORF36(ECs1197)_Seq	TGCTTGTGCCAGAGGATATG
ORF37(ECs1199)_B1F	AAAAAGCAGGCTAGTTTGGATTCTGGCTGGA
ORF37(ECs1199)_B1R	GTCAATTGTCAGTAGCGATGAGTTGTCATAATGACGCCC
ORF37(ECs1199)_B2F	CATCGCTACTGACAATTGAC
ORF37(ECs1199)_B2R	AGAAAGCTGGGTGCACCACCACTGATTTGAGA
ORF37(ECs1199)_ckF	GAAAAGGTAGGGCTGCATG
ORF37(ECs1199)_ckR	CTCTCGTCGTCGTTTCTTC
ORF37(ECs1199)_Seq	AGCAAAATCTGGCGTCGG
ORF38-40(ECs1200-1202)_B1F	AAAAAGCAGGCTCACAGTTCACCACCAGACGA
ORF38-40(ECs1200-1202)_B1R	CGGATATCACGCATCAGTCTCATTCATTGTCATGTCC
ORF38-40(ECs1200-1202)_B2F	ACTGATGCGTGATATCCG
ORF38-40(ECs1200-1202)_B2R	AGAAAGCTGGGTTCTCACCACTGAGCTAAAG
ORF38-40(ECs1200-1202)_ckF	TCAATTACACGGCTCACCTG
ORF38-40(ECs1200-1202)_ckR	GTGTTCCTTTTGGCTGAAG
ORF38-40(ECs1200-1202)_Seq	CACGACTACGGCTATGAG
ORF44(ECs1207)_B1F	AAAAAGCAGGCTCGCCGTGAATGAAGAGAGTC
ORF44(ECs1207)_B1R	GAGAGCATCAATGGTCTTTAAATGCCATGAATCCTCC
ORF44(ECs1207)_B2F	AAAGACCATTGATGCTCTC
ORF44(ECs1207)_B2R	AGAAAGCTGGGTACTCTGCGTGTCTGGGTTC
ORF44(ECs1207)_ckF	ATCAGCGATACTGGGGACTG
ORF44(ECs1207)_ckR	CGGAAATCCTGGAGACTAAAC
ORF44(ECs1207)_Seq	CGGGCAATTGCATGAGATAC
ORF45-46(ECs1208-1209)_B1F	AAAAAGCAGGCTGATGAGAACGGGGTGAATGT
ORF45-46(ECs1208-1209)_B1R	GGTTGTTTGTCGCCACTTTTCAATGGTCTTTACAAAG
ORF45-46(ECs1208-1209)_B2F	AAAGTGGCGACAAACAACC
ORF45-46(ECs1208-1209)_B2R	AGAAAGCTGGGTGTTGAGGTTCAGTCGCATC
ORF45-46(ECs1208-1209)_ckF	GGACACGACGTATTTCTGG
ORF45-46(ECs1208-1209)_ckR	AGCTCACAACATGAAAAAGGCC
ORF45-46(ECs1208-1209)_Seq	GCTGCCGCTTTAAGGTTTC
ORF47-49(ECs5606, 1210-1211)_B1F	AAAAAGCAGGCTGTATCCGGCAAACACATCAATG
ORF47-49(ECs5606, 1210-1211)_B1R	CTCCCCGTTACAGCATCATTGTCGCCACTTTTGAACC
ORF47-49(ECs5606, 1210-1211)_B2F	ATGATGCTGTAACGGGGAG
ORF47-49(ECs5606, 1210-1211)_B2R	AGAAAGCTGGGTCCGTCCATGTAAGCAAACCT
ORF47-49(ECs5606, 1210-1211)_ckF	GCCTTTATCGCTGTGATGG
ORF47-49(ECs5606, 1210-1211)_ckR	CTCAAGGGTCATCCTGAAAG
ORF47-49(ECs5606, 1210-1211)_Seq	CCAATCTTTGGGGCAATGGAAC
ORF52-53(ECs1214, ECs5607)_B1F	AAAAAGCAGGCTGTTTGGGCTGCTGACATATC
ORF52-53(ECs1214, ECs5607)_B1R	CTCTCTTTCACCCCACCAGCATATTCATCGGTTATTTCC
ORF52-53(ECs1214, ECs5607)_B2F	CTGGTGGGGTGAAAGAGAG
ORF52-53(ECs1214, ECs5607)_B2R	AGAAAGCTGGGTATCCGGGAATACAATGACGA
ORF52-53(ECs1214, ECs5607)_ckF	CAGCTGCTGGCCTTTTTCATG

ORF52-53(ECs1214, ECs5607)_ckR	GATGCATAATGACCGCTGTG
ORF52-53(ECs1214, ECs5607)_Seq	TGGACGGATAACACGAAATC
ORF56(ECs1217)_B1F	AAAAAGCAGGCTCCCGTAAAATGAGCGTGATGG
ORF56(ECs1217)_B1R	GGAATAACACCATGAAAAAATGCTCACAATAATTGCATGAG
ORF56(ECs1217)_B2F	TTTTTTCATGGTGTTATTCC
ORF56(ECs1217)_B2R	AGAAAGCTGGGTGCGAACATCCCTTTAAGTGC
ORF56(ECs1217)_ckF	CCATACGTTCCCATGAAACC
ORF56(ECs1217)_ckR	GTCAGCACGAAGTTTTGGTG
ORF56(ECs1217)_Seq	AACTCTGCGTGCTGATGTTG
ORF57(ECs1218)_B1F	AAAAAGCAGGCTGAACCGTGTTCTGTGTGTGG
ORF57(ECs1218)_B1R	CGGTTTCTTCCACCACCGACGATTCATCGCACCTGAC
ORF57(ECs1218)_B2F	CGGTGGTGGAAGAAACCG
ORF57(ECs1218)_B2R	AGAAAGCTGGGTATCCAGTCTCCCCGAATTAC
ORF57(ECs1218)_ckF	TGACGTTATCGGGACTGGTG
ORF57(ECs1218)_ckR	GGTCATTTCTGACGGCTCAT
ORF57(ECs1218)_Seq	AGCGCAGTAGCGAGTAGCAT
ORF60(ECs1221)_B1F	AAAAAGCAGGCTACAGCGTGAGGAAATGAAGC
ORF60(ECs1221)_B1R	CAGAGTTTTCAGAGCGACATATTTTTCATCAGTGAGCCATC
ORF60(ECs1221)_B2F	ATGTCGCTCTGAAAACTCTG
ORF60(ECs1221)_B2R	AGAAAGCTGGGTTCAGCGAAACGTTCAGTGAG
ORF60(ECs1221)_ckF	GGCAATGAACATCACGCTTAC
ORF60(ECs1221)_ckR	TCACTGACTTCACCGAAAGC
ORF60(ECs1221)_Seq	CGAAGGAATGAAAACGCTTC
ORF61(ECs1222)_B1F	AAAAAGCAGGCTCAGGTGGGAAGACTGTTGC
ORF61(ECs1222)_B1R	CAGTAAGTTACCCGAGCTTAAAATCCATAAATAACTCCG
ORF61(ECs1222)_B2F	AAGCTCGGGTAACTTACTG
ORF61(ECs1222)_B2R	AGAAAGCTGGGTAACCTTTTGCACGGTTCACG
ORF61(ECs1222)_ckF	GGGAGTGTATTCCGCATTTC
ORF61(ECs1222)_ckR	TGGTTGCCGTCAGGTTATTC
ORF61(ECs1222)_Seq	ACAGCGGATGAATGAAATGTC
ORF62(ECs1223)_B1F	AAAAAGCAGGCTATGCCAGTGTGGAGCAGAC
ORF62(ECs1223)_B1R	GGAAAAAAATCAGAGCTTCACAGTCGTCATTACTTCAGTTC
ORF62(ECs1223)_B2F	GTGAAGCTCTGATTTTTTCC
ORF62(ECs1223)_B2R	AGAAAGCTGGGTCAGCTCTTCTGCCTCACAC
ORF62(ECs1223)_ckF	ACGTCGTGACGGAAGATACC
ORF62(ECs1223)_ckR	TGACGACGGACATACGGTAA
ORF62(ECs1223)_Seq	CGGAACCGGTGATAATTTTG
ORF63(ECs1224)_B1F	AAAAAGCAGGCTCGATAGTGCATCTTGCTGGA
ORF63(ECs1224)_B1R	GGTTTTAATCAGTATCCCACCTTTGCCATAATTAATGACTC
ORF63(ECs1224)_B2F	GTGGGATACTGATTAAAACC
ORF63(ECs1224)_B2R	AGAAAGCTGGGTCACTTCTTCAGGCCATGTGA
ORF63(ECs1224)_ckF	TTTAACCTGGCATCCTCAGC
ORF63(ECs1224)_ckR	ACGGATCCCCTCAGAAAACT
ORF63(ECs1224)_Seq	TGCTGCAACCAATATTGACC
ORF64(ECs1225)_B1F	AAAAAGCAGGCTTCATCCGCTGTTCAAAGGTG
ORF64(ECs1225)_B1R	GTAAAAAAATCACTCAGTTCC CTCACTCATATAATTCCCCG
ORF64(ECs1225)_B2F	GGAACTGAGTGATTTTTTAC
ORF64(ECs1225)_B2R	AGAAAGCTGGGTTTGTGGGCCACTCAGGATAC
ORF64(ECs1225)_ckF	AGGACTGGAACCAGATGATG
ORF64(ECs1225)_ckR	GTATCATCGTCCTGCGTCAG
ORF64(ECs1225)_Seq	GCATTAAGTCCGGTGAGCATG
ORF65(ECs1226)_B1F	AAAAAGCAGGCTGTACGCACGGTAATGAGTC

ORF65(ECs1226)_B1R	CGTCGTCATCAGAAAAACCT TTCCGCCATGCTGTTCAGTC
ORF65(ECs1226)_B2F	AGGTTTTTCTGATGACGACG
ORF65(ECs1226)_B2R	AGAAAGCTGGGTACAGCAGTCTTCCCTTTCAC
ORF65(ECs1226)_ckF	CCGTTGATACAGCAGTGAAG
ORF65(ECs1226)_ckR	CCGGGTATGCTAACCTCAAC
ORF65(ECs1226)_Seq	GTTTCTGTTAACGGGCAGG
ORF66(ECs1227)_B1F	AAAAAGCAGGCTGTTTCCGGATGTCTGGGTTC
ORF66(ECs1227)_B1R	CAACAACACTCACACTCCACC CGTCGTCATCAGAAAAACCTC
ORF66(ECs1227)_B2F	GGTGGAGTGTGAGTGTTGTTG
ORF66(ECs1227)_B2R	AGAAAGCTGGGTGGACCTGTTTCGCCTTTAG
ORF66(ECs1227)_ckF	CCGGGTATGCTAACCTCAAC
ORF66(ECs1227)_ckR	CCTTCAGGCCTGGTATTC
ORF66(ECs1227)_Seq	TGCAGACTGGTGTTTCATGG
ORF67(ECs1228)_B1F	AAAAAGCAGGCTTCACATGGCCTGAAGAAGTG
ORF67(ECs1228)_B1R	TATTCATGTCATTCTCCTGTAACACTCACACTCCACCTCC
ORF67(ECs1228)_B2F	ACAGGAGAATGACATGAATA
ORF67(ECs1228)_B2R	AGAAAGCTGGGTATGCCTTGCATGACATTCTG
ORF67(ECs1228)_ckF	TGAGGTGAGCGTGCTTTATG
ORF67(ECs1228)_ckR	CAGCATGGATGTCACAACG
ORF67(ECs1228)_Seq	AGGACGATGATACGCCAGTT
ORF68(ECs1229)_B1F	AAAAAGCAGGCTATGGGAGCACGAGGTGAG
ORF68(ECs1229)_B1R	GCTGTCGGCTATTTCATCC AATATTCATGTCATTCTCCTG
ORF68(ECs1229)_B2F	GGATGAAATAGCCGACAGC
ORF68(ECs1229)_B2R	AGAAAGCTGGGTCCGTGACGATTTCTGGTTTGC
ORF68(ECs1229)_ckF	AAAGGCGAGAAAGGTGATCC
ORF68(ECs1229)_ckR	TGTGGCAATAGTCGCATCCG
ORF68(ECs1229)_Seq	GCCGGGAAACATTATTGAGAC
ORF69(ECs1230)_B1F	AAAAAGCAGGCTGGCCGAAGGGTGATAAGG
ORF69(ECs1230)_B1R	GAAAGCTATTCACTGTCTTCG TATTAACATGTTTATTAC
ORF69(ECs1230)_B2F	CGAAGACAGTGAATAGCTTTC
ORF69(ECs1230)_B2R	AGAAAGCTGGGTCGGAGTACGGAGTGTTAC
ORF69(ECs1230)_ckF	GCGAAAACAAAGGCAGAAGAG
ORF69(ECs1230)_ckR	CACCTGATGCAGAGCGATAA
ORF69(ECs1230)_Seq	GGAGCAGGACAGGGTAATCA
ORF70(ECs1232)_B1F	AAAAAGCAGGCTAGGCAGAACAGGAGAATGAC
ORF70(ECs1232)_B1R	GTTTTCTGGTCATACCGGCATCGGCATCACTGAAACAAAATGC
ORF70(ECs1232)_B2F	ATGCCGGTATGACCAGAAAAC
ORF70(ECs1232)_B2R	AGAAAGCTGGGTTGACTTACAGGCCTGCCTTC
ORF70(ECs1232)_ckF	TTTCAGCATTTGCAGGTCAG
ORF70(ECs1232)_ckR	CAATCACCGCGTCATAAATC
ORF70(ECs1232)_Seq	TGGGAATTCACACGAAGACA
ORF71(ECs1233)_B1F	AAAAAGCAGGCTGTTTTCGGAAGCGTATCTGC
ORF71(ECs1233)_B1R	CCTCCTGATCAGCGTAATCTTCTGGTCATACCGGCATCTC
ORF71(ECs1233)_B2F	AGATTACGCTGATCAGGAGG
ORF71(ECs1233)_B2R	AGAAAGCTGGGTACCGGAATAATGCTCACAGG
ORF71(ECs1233)_ckF	CAAAATACCGGCGAAAAACC
ORF71(ECs1233)_ckR	GGACCTTATCTATATACGGC
ORF71(ECs1233)_Seq	CGGGTGGGGATCACCATTAT
ORF72(ECs1234)_B1F	AAAAAGCAGGCTCTGGTCGGGATTTTACTGGA
ORF72(ECs1234)_B1R	GTACATAACACTTATCCCCAGAAGTATTCCAATACTCACCTCCTG
ORF72(ECs1234)_B2F	TTCTGGGGATAAGTGTTATGTAC
ORF72(ECs1234)_B2R	AGAAAGCTGGGTGAGCAGTCGCATCCTTTAC

ORF72(ECs1234)_ckF	GCTCGGTTCTTCTTGAATGG
ORF72(ECs1234)_ckR	CACTGACCCGACCATCTGTA
ORF72(ECs1234)_Seq	TATCCGGAGTGCCGTTATTC
ORF73(ECs1235)_B1F	AAAAAGCAGGCTTGAAACCCGCATTAGCACAC
ORF73(ECs1235)_B1R	CTCGCTTCAACTAATCATATAC ACCGTACATAACACTTATCCC
ORF73(ECs1235)_B2F	GTATATGATTAGTTGAAGCGAG
ORF73(ECs1235)_B2R	AGAAAGCTGGGTGGTCAGAATTTCAGGCCAATGCC
ORF73(ECs1235)_ckF	GTGTCCGAGCTGCGTAAAGAA
ORF73(ECs1235)_ckR	GTACGGGCAATAAAAAACCCG
ORF73(ECs1235)_Seq	CGTTAATGTTACGGGCGTGC
ORF74(ECs1236)_B1F	AAAAAGCAGGCTAATAAACCCGACGGGAAATC
ORF74(ECs1236)_B1R	GATACAGGTCAGAATTTCAGACTCTTCATTTAAGATATCC
ORF74(ECs1236)_B2F	CTGAAATTCTGACCTGTATC
ORF74(ECs1236)_B2R	AGAAAGCTGGGTAACACCATGAAGCCATCCTC
ORF74(ECs1236)_ckF	GTTAATGTTACGGGCGTGCT
ORF74(ECs1236)_ckR	CGCCTTTACTTCCACCCATAATG
ORF74(ECs1236)_Seq	CATCCACTGGTGCTACAGG
ORF75(ECs1237)_B1F	AAAAAGCAGGCTCGGATGATTTTGGCGTTATC
ORF75(ECs1237)_B1R	CTGTAGCAGTCAGACCGCTAT GGGTGCCATATCGGCTGAAC
ORF75(ECs1237)_B2F	ATAGCGGTCTGACTGCTACAG
ORF75(ECs1237)_B2R	AGAAAGCTGGGTCAAATATCTGGTTCGCCAGTCG
ORF75(ECs1237)_ckF	TCGGGTCAGTGGATATGAAGG
ORF75(ECs1237)_ckR	CATCTGCGCTGTACGGAGACTG
ORF75(ECs1237)_Seq	CGCTATGTCGCAAAAATCGC
ORF76(ECs1238)_B1F	AAAAAGCAGGCTCGTACAGGGCAAAAACCGTA
ORF76(ECs1238)_B1R	CTCCATAATTACAGTCTCCA TCCACCCATAATGGCCCCCT
ORF76(ECs1238)_B2F	TGGAGACTGTAATTATGGAG
ORF76(ECs1238)_B2R	AGAAAGCTGGGTCTCGTTATTTTTCTGTTGCCGG
ORF76(ECs1238)_ckF	ACCGTTTACTACCCGCTGTG
ORF76(ECs1238)_ckR	CAAAGTTCCGCTGATCAACG
ORF76(ECs1238)_Seq	ACAGCGTTGAGCGTTACCTG
ORF77(ECs1239)_B1F	AAAAAGCAGGCTTGAGTTCCATACGGCAAGG
ORF77(ECs1239)_B1R	CATATTGCCCCCTTACGCTGCCAC ATACTCCATAATTACAGTCTC
ORF77(ECs1239)_B2F	GTGGCAGCGTAAGGGGGCAATATG
ORF77(ECs1239)_B2R	AGAAAGCTGGGTCGAGATTTTCCGGGGTATTC
ORF77(ECs1239)_ckF	TGCTCAGTGCTGATGGTATC
ORF77(ECs1239)_ckR	CCTGGCCTCTTTTATCGTTTTG
ORF77(ECs1239)_Seq	GCGGATGCGTTACAGAGTTT
ORF78(ECs1240)_B1F	AAAAAGCAGGCTGCGGGTAATCTTGCTCAGTC
ORF78(ECs1240)_B1R	CACCACTCCTTAAAACAAAGAGTATCCCATATTGCCCCCTTA
ORF78(ECs1240)_B2F	TCTTTGTTTTAAGGAGTGGTG
ORF78(ECs1240)_B2R	AGAAAGCTGGGTGCTTCGGCATAAAATAACC
ORF78(ECs1240)_ckF	GAAAGGGTGGCAGGATTAC
ORF78(ECs1240)_ckR	GCATTACCGCCATTCTTGATC
ORF78(ECs1240)_Seq	AGGATAACCAGATGGCGAGA
ORF79(ECs1241)_B1F	AAAAAGCAGGCTGGCGTTTACGAAACAGCAG
ORF79(ECs1241)_B1R	CGTGAATTACTGGGCGTATCCGCTCATTCACCACTCC
ORF79(ECs1241)_B2F	TACGCCCAGTAATTCACG
ORF79(ECs1241)_B2R	AGAAAGCTGGGTACTCTGCCCCTGTGCACTTA
ORF79(ECs1241)_ckF	ATGTTGCTGCTCTCGGTTCT
ORF79(ECs1241)_ckR	ATCCATTTTCTGGCGATCAG
ORF79(ECs1241)_Seq	AAGTGACGCAGACAGACGAC

ORF80(ECs1242)_B1F	AAAAAGCAGGCTCGGAAAATCTCGATGTGCTTTC
ORF80(ECs1242)_B1R	CATTTCTGTTAATCCACTCCATAGGCCATACAGACTCC
ORF80(ECs1242)_B2F	GGAGTGGATTAACAGAAATG
ORF80(ECs1242)_B2R	AGAAAGCTGGGTGCAGGCAAAAGAACTGAAGG
ORF80(ECs1242)_ckF	GTCAAACGAAGGCTATGCAG
ORF80(ECs1242)_ckR	TGTGTCTTTTGGCGCTTTTG
ORF80(ECs1242)_Seq	ATTGCTGGAGAACGGGTATG
ORF81(ECs5608)_B1F	AAAAAGCAGGCTCGGTAATGTTGTTGGTGGTG
ORF81(ECs5608)_B1R	CTATTCGCCATTACCGTTGCTGATTAAACATAAATCGGCTCTC
ORF81(ECs5608)_B2F	CAGCAACGGTAATGGCGAATAG
ORF81(ECs5608)_B2R	AGAAAGCTGGGTTAGTGACGGCACTGGTAAC
ORF81(ECs5608)_ckF	TCTGCCTTACGGGTACAAC
ORF81(ECs5608)_ckR	GCTATGAAGCAGCAAAAGGC
ORF81(ECs5608)_Seq	CGGTAGAGCGGGATATGAAA
ORF82-83(ECs1243-1244)_B1F	AAAAAGCAGGCTCGCAGATTTACCAGGAGAAC
ORF82-83(ECs1243-1244)_B1R	GGACTATGAATGAAAAAGGTAGGTGAACATCATCCCGGTTAC
ORF82-83(ECs1243-1244)_B2F	TACCTTTTTCATTCATAGTCC
ORF82-83(ECs1243-1244)_B2R	AGAAAGCTGGGTGATGAAGAGAAGCTGCTGAAC
ORF82-83(ECs1243-1244)_ckF	TATTCCTCTGCCTTACGGGTAC
ORF82-83(ECs1243-1244)_ckR	TCTTGTTGCCGTGGAGTTTG
ORF82-83(ECs1243-1244)_Seq	GCGTATTTGCTGAAAAAGC
ORF84-85(ECs1245,ECs5683)_B1F	AAAAAGCAGGCTCAAATGGCAGCGTATTTGC
ORF84-85(ECs1245,ECs5683)_B1R	GCTCAATCCATGCTGAACACATCTGAGAAGTAAGAGACCAAG
ORF84-85(ECs1245,ECs5683)_B2F	TGTGTTCAGCATGGATTGAGC
ORF84-85(ECs1245,ECs5683)_B2R	AGAAAGCTGGGTCGTTAAGCTGGAACGGTTG
ORF84-85(ECs1245,ECs5683)_ckF	GCGGGGCTGATTAAGAAATA
ORF84-85(ECs1245,ECs5683)_ckR	TGATGACGGTCGCACTTGTG
ORF84-85(ECs1245,ECs5683)_Seq	CGCTACTGTTGAACGCTCTG
ORF86-90(ECs1246-1250)_B1F	AAAAAGCAGGCTTAGCAAGCTGGGACGGTTAC
ORF86-90(ECs1246-1250)_B1R	GGAAAGGAAAATGGCAGATGTGAATCAATGACCTGGCCTG
ORF86-90(ECs1246-1250)_B2F	ATCTGCCATTTTCCTTTCC
ORF86-90(ECs1246-1250)_B2R	AGAAAGCTGGGTATCGCATACTGACCGCACTG
ORF86-90(ECs1246-1250)_ckF	TGAAGGACGGGGTTTTACAG
ORF86-90(ECs1246-1250)_ckR	TGAGGCATTTACCGTTACCC
ORF86-90(ECs1246-1250)_Seq	GTGTTCAGCATGGATTGAGC
ORF91(ECs1251)_B1F	AAAAAGCAGGCTCAACTCTCAGCTTCCCAACC
ORF91(ECs1251)_B1R	GGAGGTCATCATGACCAGTTTCGAATTCTAAAACCAAAG
ORF91(ECs1251)_B2F	ACTGGTCATGATGACCTCC
ORF91(ECs1251)_B2R	AGAAAGCTGGGTGACCCGCGTTTGGTAATATC
ORF91(ECs1251)_ckF	CTTACGAGTTCGGCTTCCAG
ORF91(ECs1251)_ckR	GCTGTGCTGTCCACCTTTTT
ORF91(ECs1251)_Seq	AGCTCTGCATCCACGAACAG
B1F (Added to respective ORF primres)	AAAAAGCAGGCT
B1R (Added to respective ORF primres)	AGAAAGCTGGGT
M13F	CGTTAACGCTAGCATGGATCTC
M13R	ACATCAGAGATTTTGAGACAC
pABB-CRS2_ckF	CGGCAGGTATATGTGATGGG
pABB-CRS2_ckR	CACATGTGGAATTGTGAGCGG
**[b]For constructing the plasmids for complementation assays**	
ORF60(ECs1221)_F	GGC**<u>GAATTC</u>**CACACACTGGATGGCTCACTG
ORF60(ECs1221)_R	GGC**<u>GTCGAC</u>**TTTTCAGAGCGACATTTCATTC

ORF61(ECs1222)_F	GGC**GAATTC**ACCATGCGGAGTTATTTATGG
ORF61(ECs1222)_R	GGC**GTCGAC**TTACCCGAGCTTCTCCAGAAG
ORF62(ECs1223)_F	GGC**CCCGGG**ACTGAAGGAGAACTGAAGTA
ORF62(ECs1223)_R	GGC**GTCGAC**AAATCAGAGCTTCACTGCTG
ORF65(ECs1226)_F	GGC**GAATTC**CGTGTTGCGGAGACTGAACAG
ORF65(ECs1226)_R	GGC**GTCGAC**TCATCAGAAAAACCTCTGCCTG
ORF67(ECs1228)_F	GGC**GAATTC**AGTGTTTAACGGAGGTGGAGTG
ORF67(ECs1228)_R	GGC**GTCGAC**ATGTCATTCTCCTGTTCTGCCTG
ORF70(ECs1232)_F	GGC**CCCGGG**TCAGAATGTCATGCAAGGCATTTTG
ORF70(ECs1232)_R	GGC**GTCGAC**TGGTCATACCGGCATCTC
ORF71(ECs1233)_F	GGC**GAATTC**AGGAGGTAAATGAAGAGTATAGCAAC
ORF71(ECs1233)_R	GGC**GTCGAC**TCAGCGTAATCTGCCTATG
ORF79(ECs1241)_F	GGC**GAATTC**CTTTGTTTTAAGGAGTGGTGAATG
ORF79(ECs1241)_R	GGC**GTCGAC**TTACTGGGCGTAATTCTG
ORF80(ECs1242)_F	GGC**GGATCC**CGGCCATTGTGCCGGGTTTTTTTATG
ORF80(ECs1242)_R	GGC**GTCGAC**CTGTTAATCCACTCCCACTTTTTTG

Note: [a] These primers and probe were used to quantify a chromosomal backbone (CB) region.

[b] Restriction sites are indicated by bold letters and underlining.
